# Patient-Specific 3D-Printed Osteotomy Guides and Titanium Plates for Distal Femoral Deformities in Dogs with Lateral Patellar Luxation

**DOI:** 10.3390/ani14060951

**Published:** 2024-03-19

**Authors:** Enrico Panichi, Fulvio Cappellari, Ekaterina Burkhan, Gaetano Principato, Marco Currenti, Marco Tabbì, Francesco Macrì

**Affiliations:** 1 Centro Traumatologico Ortopedico Veterinario, Via C. Festa 9, 16011 Arenzano, Italy; enricopanichi76@gmail.com (E.P.); fulvio.cappellari@ctovet.com (F.C.); gaetanoprincipato@gmail.com (G.P.); marcocurrenti.vet@gmail.com (M.C.); 2 Bonabyte, Begovoy Proezd 7, 125284 Moscow, Russia; k.burkhan@bonabyte.net; 3 Department of Veterinary Sciences, University of Messina, via Palatucci Annunziata, 98100 Messina, Italy; francesco.macri@unime.it

**Keywords:** complex femoral deformities, lateral patellar luxation, virtual surgical planning (VSP), computer-aided design (CAD), patient-specific 3D-printed osteotomy guides, patient-specific 3D-printed fixation plate, distal femoral trapezoid osteotomies (DF-TO)

## Abstract

**Simple Summary:**

This study describes the use of virtual surgical planning and three-dimensionally printed patient-specific guides and implants for the correction of three femoral deformities via distal femoral osteotomy in two patients with grade IV lateral patellar luxation. The use of computer-aided design software reported in this study was simple and allowed for the assessment of the degree of deformity and for the planning and simulating of the corrective osteotomy. The procurvatum was also corrected in all femurs to increase knee extension. After the surgical correction the preoperative, expected and postoperative measurements were compared to assess the efficacy of the correction performed. In all cases, weight bearing was observed two days after surgery. Virtual surgical planning combined with the use of patient-specific three-dimensionally printed osteotomy guides and titanium plates led to optimal outcomes in both patients. Patient-specific osteotomy guides reduced intraoperative time, eliminated the need for intraoperative diagnostics and ensured a more accurate osteotomy. Patient-specific titanium plates reduced surgical complications and allowed for shorter postoperative recovery times. The use of virtual surgical planning and three-dimensionally printed patient-specific instruments for distal femoral osteotomy and subsequent plate fixation ensured accurate correction of femoral deformities with good clinical outcomes.

**Abstract:**

The aim of this study was to describe the diagnosis and treatment of grade IV lateral patellar luxation (LPL) in two adult large breed dogs with complex femoral deformities using patient-specific three-dimensionally (3D) printed osteotomy guides and implants. Computed tomography (CT) scans were obtained for virtual surgical planning (VSP) using computer-aided design (CAD) software, which allowed for 3D reconstruction and manipulation of the femoral deformities, providing a preoperative view of the correction. Of the two patients, one was affected bilaterally and the other unilaterally, but both dogs were from the same litter. Therefore, the healthy femur of the unilaterally affected patient was used as the physiological reference for the virtual surgical correction. Three distal femoral trapezoid osteotomies (DF-TO) followed by reduction and internal fixation with plates were performed using patient-specific 3D-printed osteotomy guides and implants. This type of osteotomy permitted correction of procurvatum in all the femurs to increase knee extension, raise the dog’s lumbar spine and correct the kyphosis. Preoperative, expected and postoperative femoral angles were compared to evaluate the efficacy of virtual surgical planning and the outcome of surgical correction. Radiographic follow-up, passive range of motion and functional recovery were recorded. There were no major complications requiring revision surgery. Significant clinical improvement was observed in both patients. This study suggests that the treatment used represents a viable surgical alternative to restore limb alignment in patients with complex femoral deformities.

## 1. Introduction

Patellar luxation (PL) is one of the most common orthopedic conditions in dogs. Lateral patellar luxation (LPL) is less common than medial patellar luxation (MPL) and is more commonly diagnosed in large or giant breeds than in small breeds [1,2,3,4,5]. The predisposition of these breeds, the high prevalence of bilateral disease and the frequent association with congenital developmental abnormalities suggest a hereditary component [6,7,8,9]. Although the pathogenesis remains unclear, PL is often associated with malalignment of the quadriceps muscles, patella and patellar tendon, as well as femoral and tibial deformities. Malalignment of the extensor (or quadriceps) mechanism of the stifle, which consists of the quadriceps muscle group, patella, patellar tendon and ligament, trochlear groove and tibial tubercle, appears to play a key role in the development of this pathology. Any abnormality in the components of this mechanism can lead to anatomical changes in the distal femur and proximal tibia during growth, resulting in patellar instability. In turn, primary PL can lead to secondary bone changes. It has been suggested that hypoplasia of the vastus medialis muscle may be the initiating factor that causes other deformities leading to lateral patellar luxation, in addition to abnormal angles of inclination and anteversion [1,8]. Bone deformities are complex musculoskeletal pathologies that lead to varying degrees of PL during growth, with the inability to extend the leg joint and lameness. If not properly treated, the condition progresses to osteoarthritis, causing significant discomfort, pain and disability in affected animals [1,8,9,10]. Grade IV represents the most severe form of PL and is characterized by a permanent dislocation of the patella that cannot be manually replaced in the femoral groove, persistent lameness and abnormal posture. Bilateral dislocation causes compensatory antalgic kyphosis of varying degrees. In lateral dislocation, the stifles appear to be close together while the distal extremities are abducted (valgus) [1,10]. Several surgical techniques have been reported to correct patellar luxation. Distal femoral osteotomy (DFO) is one of the most performed surgical techniques to correct grade IV patellar luxation secondary to femoral deformity, often in combination with other procedures [1,2,3,4,5,11,12,13,14,15,16,17].

Virtual surgical planning (VSP) and three-dimensionally (3D)-printed patient-specific instruments (PSIs) are widely used in human medicine to correct limb deformities and have been described in veterinary medicine in recent years [18,19,20,21,22,23,24]. VSP uses cross-sectional images of the affected area and computer-assisted planning software to plan, simulate and validate the surgical procedure to correct the deformity, helping the surgeon to implement it in the subsequent surgical phase [25]. Patient-specific instruments (PSI) produced via three-dimensional (3D) printing include patient-specific guides (PSGs) and plates (PSPs), both of which are designed from computed tomography (CT) scans based on the anatomical, geometric and biomechanical characteristics of the recipient. Unlike generic cutting guides, which are designed based on average reference values, PSGs allow for customized bone cuts, reducing the use of intra-operative diagnostics and operating time, increasing accuracy of bone cuts and allowing for precise correction of complex deformities. In veterinary medicine, the use of PSGs has been described in the treatment of numerous orthopedic conditions, both spinal and appendicular, including corrective osteotomies for tibial and femoral deformities [25,26,27,28,29,30,31,32,33,34,35,36,37,38,39,40,41,42]. The aim of this study was to describe the restoration of alignment in the sagittal, frontal and transverse planes in two patients with grade IV LPL treated with distal femoral trapezoid osteotomies (DF-TO) using VSP and patient-specific 3D-printed osteotomy guides and titanium plates.

## 2. Case Description

### 2.1. Animals and Study Design

Two nonspayed three-year-old American Bulldog males from the same litter, both suffering from grade IV LPL, were included in this study. Dog 1 had a bilateral deformity, weighed 44 kg and presented severe kyphosis and bilateral hindlimb lameness. Dog 2 had a unilateral deformity, weighed 45 kg and presented mild kyphosis and lameness of the right limb. In addition, both patients had decreased range of motion in extension, although this was greater in dog 1. Both subjects were referred for persistent lameness and abnormal posture that had been present for over two years. Both owners reported that from the age of 6 months, the subjects exhibited an abnormal ‘crouched’ gait with the stifles close to each other and the distal extremities abducted (valgus). The diagnosis of grade IV LPL was made one year prior to presentation, but at that time both owners refused corrective surgery.

### 2.2. CT Scan Acquisition and Deformity 3D Measurements

Both patients were sedated with a combination of intravenous dexmedetomidine (Dexdomitor, Vétoquinol Italia S.r.l., Bertinoro, Italy; 0.005 mg/kg) and butorphanol (Dolorex, MSD Animal Health S.r.l, Milan, Italy; 0.3 mg/kg) and underwent pelvic limb computed tomography (CT) using a 16-slice helical scanner (Somatom Emotion 16; Siemens, Germany) to identify and characterize deformities by taking preoperative measurements in three different planes of the femur and tibia.

**Figure 1:**
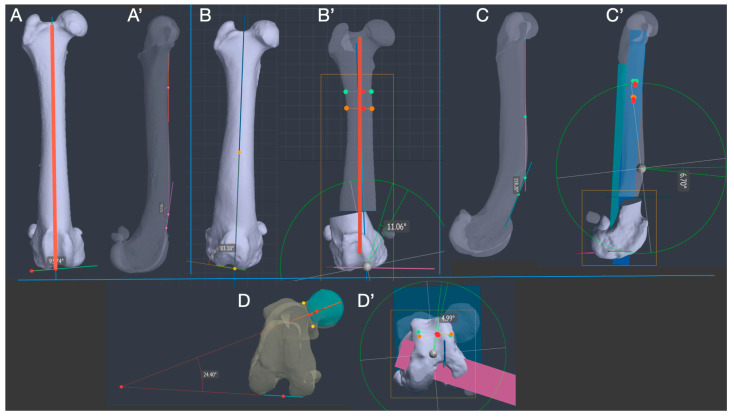
Measurement and planning in all three plane of deformity correction of the distal femur. The normal femur of Dog 2 was used as a template (**A**,**A’**). Valgus (**B**,**B’**), procurvatum (**C**,**C’**) and torsion (**D**,**D’**) deformities was present at the same time. Because of the juxta-articular CORA, an osteotomy was performed proximally and the ACA was based on CORA (**B’**). The anatomic axis of the femur was restored via translation of the distal segment (red line) (Osteotomy rule n.2). (BonaPlanner computer-aided design CAD software version 1.0.0 © Bonabyte LLC, Moscow, Russia).

DICOM (digital imaging and communications in medicine) CT images were reconstructed to a slice thickness of 0.6 mm using a bone tissue algorithm and exported to the BonaPlanner computer-aided design (CAD) software (©Bonabyte LLC, Moscow, Russia), from which a 3D reconstruction of the limb was obtained via a high-quality segmentation process supervised by a bioengineer. The 3D reconstruction allowed for manipulation of the limb in the sagittal, frontal and transverse planes and the measurement of preoperative values of anatomical lateral distal femoral angle (aLDFA), anatomical cranial distal femoral angle (aCrDFa), femoral torsion angle (FTA), mechanical medial proximal tibial angle (mMPTA) and mechanical medial tibial angle (mMDTA) (Figure 1).

These values were used in the subsequent virtual surgical planning (VSP) of the femoral osteotomy to calculate expected values and for comparison with the postoperative measurements obtained. Measurements in the sagittal, frontal and transverse planes of the tibia showed no changes or deformities in either patient. In contrast, a common deformity characterized by excessive valgus, procurvatum and a slightly increased femoral anteversion was observed in all pathological femurs.

### 2.3. Virtual Surgery, Patient-Specific Osteotomy Guide and Titanium Plate Design

**Figure 2:**
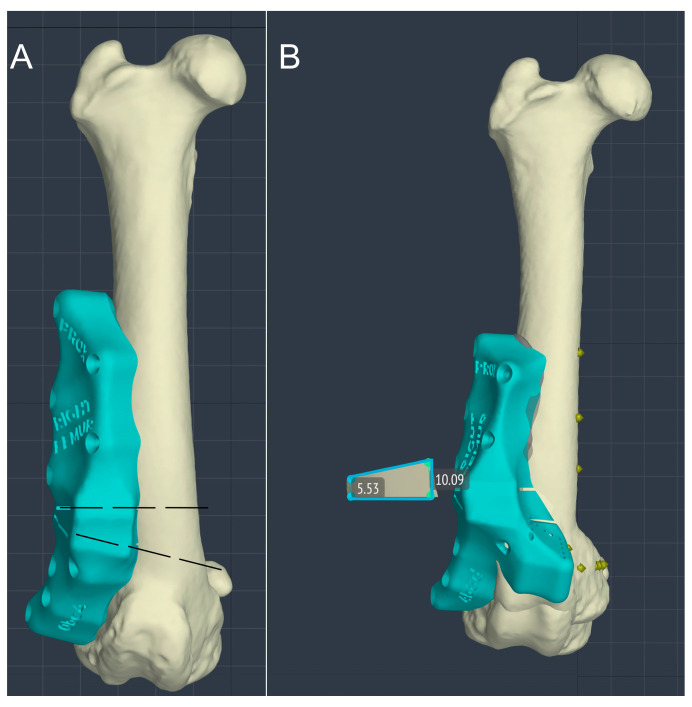
Virtual surgical simulation of the femoral osteotomy with a frontal view of the femur and lateral application of the osteotomy guide. The dashed osteotomy lines (**A**) show the planes through which the oscillating blade passed to perform the osteotomy. Result of the femoral osteotomy with removal of the trapezoid bone segment (**B**).

After determining the extent of the deformity, the CORA (center of rotation of angulation) and ACA (angulation correction axis) were traced and the correction simulated using CAD software. The distal femoral trapezoid osteotomy (DF-TO) and subsequent virtual reduction were performed using the software, which allowed for visualization of the femur in three planes and for the manipulation of the two bone segments postosteotomy. The desired result of limb alignment was observed, measured and recorded. In the patient with unilateral femoral deformity (Dog 2), the contralateral limb was used as a model for both dogs as they were from the same litter. Corrections were made in the frontal and sagittal planes based on the alignment of the normal femur. However, as the 16° FTA angle of the healthy limb differed from the normal CT values reported in the literature (19.6 ± 7.9) [43], it was corrected to aid 20° of anteversion angle to avoid the risk of iatrogenic MPL in case of torsional overcorrection of the femur. A distal femoral trapezoid osteotomy (DF-TO) was chosen to simultaneously correct the valgus, procurvatum and torsional deformities of the femur. However, whereas a standard CWO would have involved a medial-lateral approach, in our study, thanks to the VSP technique, we decided to integrate the corrective osteotomy with a femoral shortening. This allowed a lateral approach and created a trapezoidal rather than a wedge-shaped bone segment (Figure 2).

Treatment of grade IV LPL and realignment of the quadriceps mechanism and underlying bony deformities corrected the compensatory antalgic kyphosis due to reduced passive range of motion (PROM) in knee extension. As a juxta-articular deformity had to be corrected, Paley’s rule number 2 was used to achieve alignment of the limb and create the necessary space for the plate application [44]. The ACA rotation point was positioned at the level of the CORA, but the osteotomy site was positioned above the CORA to allow sufficient space to stabilize the distal segment with four plate screws. Lateral translation of the distal segment in the frontal plane was accepted to align the mechanical axis of the limb. The anatomical distal lateral femoral angle (aLDFA), procurvature angle (PROC) and femoral torsion angle (FTA) were recorded and used for comparison with preoperative measurements to evaluate the accuracy of the virtual correction.

The stereolithographic (SLA) 3D printing technique was used to produce the osteotomy guides in Nextdent Dental SG material. The osteotomy guides were designed with 3D contours that accurately replicated the shape of the bone, ensuring high precision in the positioning and angles of the cutting instruments to ensure the best possible fit of the customized implants and the stability of the osteotomy site. Similarly, a titanium plate was designed to allow internal fixation of the osteotomy site and bone healing once alignment was achieved. The patient-specific titanium implants were manufactured using powder bed additive manufacturing (PB-AM) techniques via electron beam melting (EBM) from the Ti-6Al-4V alloy specified by ASTM and US FDA standards [23,35] for surgical implants.

Screw location and diameter were selected by the surgeon to have at least 4 screws distally and 5 screws proximally. The surgeon decided to plan a plate with a polyaxial position of the screws to ensure implant stability as in a type 1b external skeletal fixation cranio-lateral to caudo-medial and latero-medial. The location of the screws was adjusted based on the analysis of the bone condition and its density revealed by the bioengineer during the segmentation of the CT images, ensuring the reliability of the fixation.

The shape of the plate was suggested by the surgeon to use the lateral surgical approach to the femur. The thickness and width of the plate were chosen by the bioengineer in relation to the patient’s size and depended on the position, type and diameter of the previously planned screws. Plate thickness was greatest at the level of the osteotomy and thinnest distally near the joint where soft tissue coverage was least.

**Figure 3:**
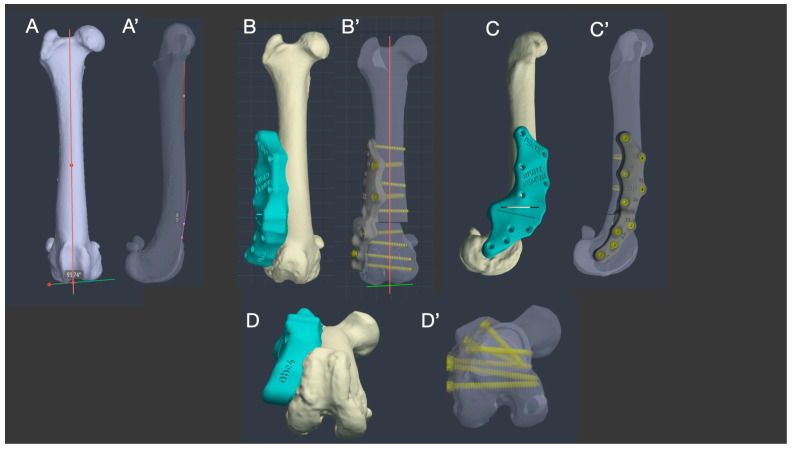
Virtual surgical simulation of distal femoral osteotomy and fixation with patient-specific 3D-printed guides and plates. The normal femur of Dog 2 was used as a template (**A**,**A’**). Simultaneous correction of valgus (**B**,**B’**), procurvatum (**C**,**C’**) and anteversion (**D**,**D’**) was achieved. A 1 cm femoral shortening was planned to avoid additional muscle tenotomy of the quadriceps muscle, which was affected by a severe contracture. (BonaPlanner computer-aided design CAD software version 1.0.0 © Bonabyte LLC, Moscow, Russia).

The cutting guides were made based on the saw blade parameters provided by the surgeon for the planned ostectomy and the generally accepted drill bit diameters used to place screws of the selected diameter (Figure 3).

### 2.4. Surgical Technique

For both patients, the anesthetic protocol included premedication with acepromazine (Prequillan, Fatro S.p.A., Bologna, Italy; 0.02–0.04 mg/kg) and methadone (Semfortan, Eurovet Animal Health B.V., Bladel, The Netherlands; 0.2 mg/kg), induction with propofol (Propovet Multidose, Zoetis S.r.l., Rome, Italy; 4 mg/kg EV) and maintenance with sevoflurane (SevoFlo, Zoetis Belgium SA, Louvain-la-Neuve, Belgium). Intraoperative analgesia was achieved with fentanyl (Fentadon, Eurovet Animal Health B.V., Bladel, The Netherlands; 2–4 µg/kg IV) at a constant infusion rate. Antibiotic treatment with cefazolin (Cefazolin TEVA, Milan, Italy; 20 mg/kg) was administered intravenously 30 min before surgery and repeated every 90 min until the end of the procedure. During anesthesia, electrocardiography, heart and respiratory rate, end-tidal CO_2_, blood O_2_ saturation and noninvasive blood pressure were recorded.

**Figure 4:**
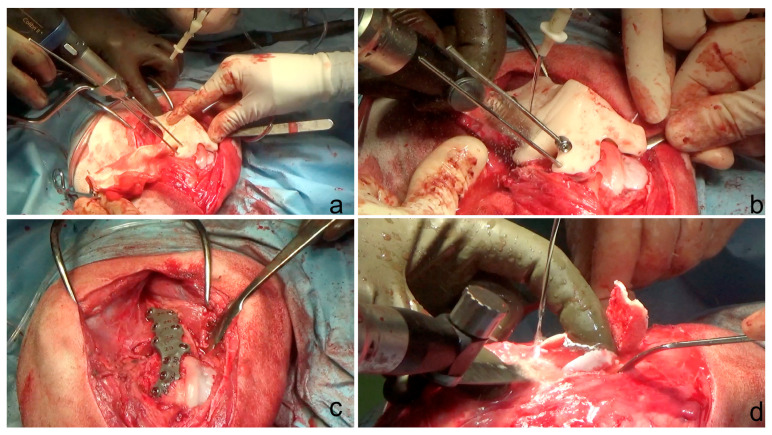
(**a**) Surgical custom guide positioning by 2.5 mm K-wire; (**b**) ostectomy performed with an oscillating saw; (**c**) 3.5 mm custom locking plate application. The planned screw length was engraved on the surface of the plate for each of the holes; (**d**) block recession sulcoplasty.

After induction of general anesthesia, the dogs were placed in dorsolateral recumbency and a hanging limb technique was used for scrubbing and surgical draping. A standard lateral approach to the distal femur and stifle and a craniolateral arthrotomy were performed to assess the depth of the trochlear sulcus and the appearance of the articular cartilage, cranial cruciate ligament and meniscus. After joint inspection, the patient-specific 3D-printed femoral osteotomy guide was placed in position and secured to the bone with 2.5 mm K-wires of the same diameter as the guide holes (Figure 4). With the guide correctly positioned, two osteotomies were performed parallel to the cutting guide lines surfaces (Figure 4). Once the osteotomies were completed, the guide was removed. The trapezoidal bone segment was removed and reduction was achieved by placing the 3D-printed titanium plate with the 3.5 mm titanium screw inserted in the previously drilled holes for the guide fixation (Figure 4). Trochlear block recession, lateral desmotomy and medial imbrication of the retinaculum were performed to improve patellar stability (Figure 4). The surgical site was flushed with 2 L of sterile saline solution and the soft tissues were sutured routinely. In dog 1, the DFOs were performed in double stage with an interval of three weeks between the procedures. The duration of surgery (mean ± SD) was 75 ± 10 min.

### 2.5. Postoperative Care

**Figure 5:**
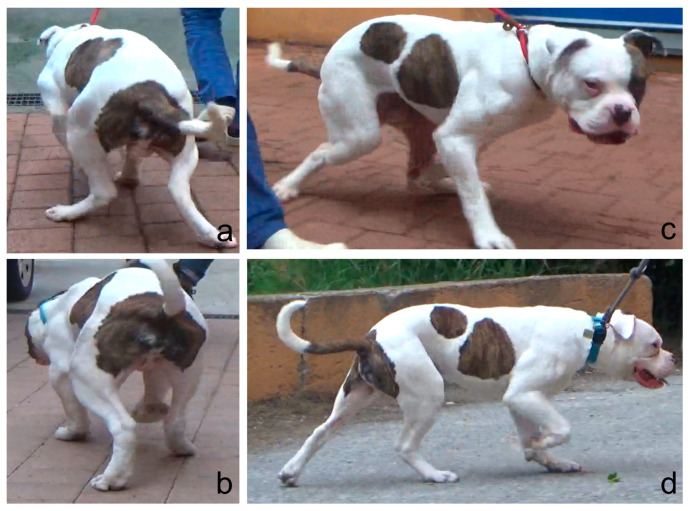
Dog 1: (**a**) preoperative view from behind; (**b**) postoperative view from behind; (**c**) preoperative lateral view; (**d**) postoperative lateral view; (**a**,**b**) a bilateral valgus conformation of the stifles with consequent external rotation of the foot and severe lumbar spine kyphosis because of the lack of the extension of both knees is visible. (**c**,**d**) Improvement of the extension led to a resolution of the lumbar kyphosis.

A supportive soft bandage was applied to the operated limb and maintained for 48 h to limit postoperative swelling [45]. Both patients were hospitalized for observation and postoperative supportive care. Patients were discharged 24 **h** after surgery, once they were toe touching on the operated limb without clinical signs of pain. During the first 24 h, postoperatively, each dog received antibiotic treatment with cefazolin (20 mg/kg, EV, q12h) and analgesic treatment with methadone (0.2 mg/kg, IM, q12h). Then, both antibiotic and analgesic treatment were replaced by oral administration of cefadroxil (Cefa-Cure Tabs cpr, MSD Animal Health S.r.l., Milan, Italy; 20 mg/kg, q24h for 7 days) and tramadol (Altadol, Formevet S.r.l., Milan, Italy; 2–4 mg/kg, q8h), respectively. Postoperative analgesia was adapted to the clinical course and needs of each patient. In addition, oral anti-inflammatory therapy with meloxicam (Metacam, Boehringer Ingelheim Animal Health Italia S.p.A., Noventana, Italy; 0.2 mg/kg on the first day, then 0.1 mg/kg, q24h for 10 days) and gastroprotective therapy (omeprazole at 1 mg/kg and sucralfate at 50 mg/kg, both OS for 15 days) were administered. During the first postoperative week, cooling therapy with ice was used five times a day for 10 min per session to reduce initial oedema and wound inflammation [45,46,47]. Owners were given clear and strict instructions to follow during the postoperative period, such as keeping the patient in a confined space and avoiding sudden exertion or jumping from any height. Walking was limited to five short sessions on the lead, each lasting 10–15 min. In the second postoperative week, a plan of rehabilitative stretching exercises for the quadriceps muscles was introduced to improve PROM, reduce postoperative pain, restore normal function, regain muscle strength and achieve a normal gait pattern. Physiotherapy was continued for 6 months with resolution of lumbar kyphosis and lameness in both patients (Figure 5).

### 2.6. Postoperative CT Scan, X-rays and Joint Angle Measurements

Immediately after surgery, a clinical evaluation of the position of the patella in the trochlear groove was performed to assess the presence of residual dislocation and radiographs were taken in both standard orthogonal projections to assess the correct positioning of the plate. In addition, a CT scan and joint angle measurements were performed to assess the accuracy of the surgical technique and the degree of correction achieved. Joint orientation angles and the degree of deformity in the sagittal, frontal and transverse planes were compared between preoperative CT images, CAD images of the planned virtual correction and postoperative CT images (Table 1).

Clinical follow-up was performed at 1, 3, 6, 18, 24 and 36 months to assess thigh circumference, PROM, limb alignment and potential complications (Table 2).

Radiographic follow-up was performed at 3 and 6 months to assess bone healing.

Telephone follow-up confirmed that both dogs were weight-bearing on the affected limb within 2 days of surgery and throughout the entire postoperative period.

At the 1-month follow-up, patellar instability in full flexion similar to residual grade I LPL persisted in both dogs, probably due to the presence of knee swelling. Despite this, a marked improvement compared to preoperative limb use was observed and a gradual increase in activity and continuation of PROM exercises was encouraged.

**Figure 6:**
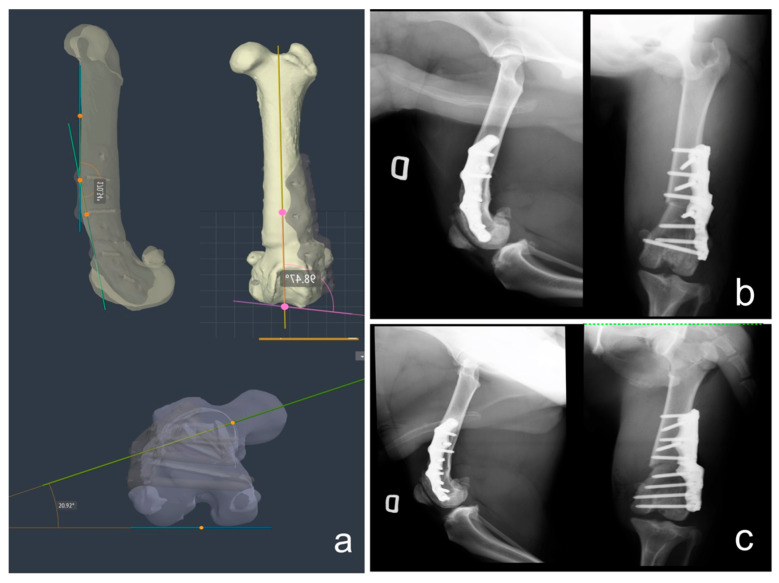
Dog 1: (**a**) postoperative CT scan imaging after segmentation; (**b**) 6-month RX follow-up; (**c**) 18-month RX follow-up.

At the 3-month follow-up, resolution of knee swelling and complete muscle realignment resulted in resolution of the instability present at the previous follow-up and a marked improvement in PROM compared with preoperative values. Radiographs confirmed complete bone healing in both patients (Figure 6). The implants were stable in both dogs. Loosening of a distal screw of the plate was observed in dog 2, but callus formation was complete and not associated with fistulae or clinical signs of pain. No complications were observed in dog 1.

PROM values remained constant at the 6-month follow-up. Radiographs confirmed bone healing and implant stability in both patients. At the 18-month clinical follow-up, lameness and decreased thigh circumference were noted in the left limb of dog 1 and in the right limb of dog 2. Radiographs showed loosening of one screw and knee effusion in both patients. The implants were therefore removed with the consent of the respective owners. No complications were noted in the contralateral limb of dog 1, with complete osteointegration of the plate. After removal of the implants, progressive clinical improvement was observed in both patients, which was confirmed up to the last follow-up at 36 months.

## 3. Discussion

This study described the use of VSP and 3D-printed patient-specific guides and implants for the correction of limb alignment via distal femoral osteotomy (DFO) in two patients affected by grade IV LPL.

In veterinary medicine, the use of 3D stereolithography has been reported in the literature for planning the correction of complex limb deformities or for fracture treatment [25,26,27,28,29,30,31,32,33,34,35,36,37,38,39,40,41,42]. Optimal surgical management requires accurate characterization of the deformity, adequate preoperative planning and careful and precise intraoperative execution. Characterizing the deformity means determining the position, extent and direction of the malalignment and it is essential for proper preoperative planning. Correction of limb deformities is traditionally performed using the CORA method, based on orthogonal radiographs. The greater the degree and complexity of the deformity, the more difficult preoperative planning becomes, especially when using 2D radiographs [44,48,49,50,51,52]. Computed tomography (CT) is more suitable than radiography for the diagnosis and characterization of complex femoral deformities [53,54]. In addition, DICOM images obtained from CT scans can be exported to CAD software which allows for an accurate assessment of the deformities, a simulation of the surgery by means of virtual preoperative planning (VSP) of the corrective osteotomy and finally the 3D printing of bone models or patient-specific instruments (PSI) [23,24,25,35]. Resection planning, screw position and diameter selection, virtual ostectomy and bone segment movement operations and all the necessary measurements were performed using Bonabyte software. The use of CAD software reported in this study was simple and made it possible to visualize the bone in all planes (sagittal, frontal and transverse) to assess the degree of deformity, to plan and simulate the corrective osteotomy by calculating the exact size of the bone segment and, finally, to re-evaluate the bone after the surgical correction to compare the preoperative, expected and postoperative measurements to assess the efficacy of the correction performed. The ability to quickly change the standardized views (sagittal, frontal and transverse) allowed for an easy understanding of the effect of the planned surgery. During the VSP and 3D design of the PSI, the surgeon’s role was to draw the shape of the customized guide on the bone model using a brush tool and to indicate the specific anatomical landmarks to be considered in relation to the chosen surgical approach. For the osteotomy guide, we considered the proximal part of the trochlea to be an easily recognizable landmark to have a guide that would adhere tightly to the bone and avoid extensive soft tissue dissection. A bioengineer then designed the osteotomy guide and the surgeon approved the final design. For the plate design, the surgeon chose the number, location and direction of the screws in the proximal and distal bone segment. For the latter, the software used included 3D models of cortical and locking screws of various length and thicknesses. At this stage, the role of the bioengineer was particularly important in the design of patient-specific implants, with technical suggestions on biomechanics such as plate thickness. Part of the VSP was the possibility of performing a trial operation on 3D-printed bone models, allowing the guides and plates to be tested prior to surgery [26,27]. However, we chose to omit this step, which would have involved an additional cost to the owners.

The lack of breed-specific reference values for normal joint angles makes it difficult to identify the target values for calculating the correction [54]. While the contralateral limb may be the most appropriate reference for alignment in dogs with unilateral anomalies, dogs with anomalies such as PL often have both femurs deformed. In the absence of breed references, the joint angles of a specific bone can be calculated using the anatomical or mechanical axes as defined by Dudley et al. [43] and Paley [44]. Another alternative would be to use as reference values those of a breed as close as possible in size and conformation to that of the patient to be corrected. In our case, for example, a good starting point would have been to use the values reported for the Rottweiler [55]. In this study, the healthy femur of dog 2 was used as a template for both the pathological femur of the same patient and the two femurs of dog 1, as they belonged to the same breed and litter. Due to the juxta-articular deformity, the CORA was located very close to the joint and for this reason we chose the osteotomy rule number 2. This meant that we made the osteotomy proximal to the site of the deformity to have enough space to put at least three screws in the distal segment. As a result, we restored the normal axis of the femur, but with translation of the bony apex at the level of the osteotomy. After the desired correction, the anatomical axis was exactly in the center of the joint, as in the normal femur used as a model (Figure 1).

All affected femurs in our study had a procurvatum deformity. This was corrected with the aim of indirectly improving the extension angle of the knee. Joint motion of the knee occurs in the sagittal plane and can therefore be influenced by correction of the underlying tibial or femoral deformity. Correction of the sagittal plane and restoration of the knee extension angle can significantly improve the outcome in the treatment of severe cases of lateral patellar luxation, especially when associated with a fixed flexion deformity causing lumbar dorsal kyphosis and helps to solve the latter [44]. Based on the accuracy and stability achieved, the authors concluded that the surgical approach used in this study was superior to other DFO methods, such as open wedge correction, for complex deformities such as those reported here. Due to the direct contact between the bone segments, the closed wedge osteotomy promoted load sharing between plate and bone, reducing the risk of implant failure and accelerating bone healing time. An open wedge osteotomy, regardless of the precision in the execution and realignment of the limb, would not have had the same level of stability due to the lack of bone apposition, increasing the risk of catastrophic implant failure. The latter would probably affect the speed of healing, which, similar to what has already been suggested in the literature, we can reasonably assume would have been longer [15]. In addition, the open wedge osteotomy would have increased the length of the femur, which could have led to further muscle contracture.

The other options available in cases of reduced knee joint extension or contracture of the quadriceps mechanism are the muscle release procedures reported in the literature [10]. Soft tissue anomalies that are often associated with grade IV patellar luxation such as joint contracture, joint laxity and severe quadriceps contracture also need to be understood and addressed when correcting sagittal plane deformity. The permanence of the condition over time result in a more severe contracture in adults than in young dogs less than one year of age. Both cases described were adult subjects with significant chronic muscle contracture, absence of trochlear ridge and inability to reduce the laterally dislocated patella due to the laterally and noncranially dislocated quadriceps muscle. For this reason and based on the surgical team’s personal experience with the surgical treatment of LPL of IV, femoral shortening was preferred to the muscle release procedures. To address the patella within the trochlea and restore the length relationship between the femur and the quadriceps muscle, we decided to shorten the femur by 1 cm of diaphyseal bone. This technique was chosen to avoid an additional tenotomy of the rectus femoris muscle, which can lead to increased morbidity during the recovery period. In addition, integrating the femoral shortening into the corrective osteotomy with VSP allowed for a lateral approach instead of the standard medial-lateral one. As a result, the osteotomy created a trapezoidal rather than a wedge-shaped bone segment. Based on this experience and these considerations, the authors recommended early treatment (within the first year of life or before completion of somatic growth) to avoid complications such as chronic soft tissue contracture, stifle joint anchylosis and periarticular fibrosis.

The postoperative angles were consistent with those of the virtual surgery in sagittal and transverse plane, but the frontal plane showed a mild overcorrection of the valgus deformity which resulted in a varus deformity of less than 5 degrees. This discrepancy between the expected and obtained measurements demonstrates a loss of primary reduction during plate application. The presence of fibrous tissue on the lateral aspect of the condyle can cause suboptimal plate placement resulting in under/overcorrection of limb alignment. We learnt that the use of a patient-specific 3D plate must match the bone surfaces without inconsistencies and this can be obtained via a meticulous debridement of the soft tissue covering the bone surface before the application of both guide and plate. Furthermore, the presence of soft tissue such as articular cartilage covering the distal subchondral bone can adversely affect the placement of a reduction guide and reduce the accuracy of the femoral osteotomy and reduction [28].

The use of a reduction guide has been described in the literature after the bone has been cut with a cutting guide and before plate application [25,26,27,28,29]. The use of this additional guide increases the cost of the surgery and requires a wider surgical approach to allow its use and the simultaneous application of the plate. In our case, no reduction guide was used and the reduction was performed directly by applying the plate. The choice to avoid the use of a reduction guide allowed for the limitation of the size of the surgical approach, making it smaller and less invasive and avoided overly aggressive soft tissue manipulation. Despite the limited surgical approach, no difficulties or errors were encountered with either the osteotomy guide or the plate placement. Two of the three plates were removed due to lameness that developed 18 months after surgery. For financial reasons, we decided to remove the implants only if there was lameness in the operated limbs. The only remaining implant was completely osteointegrated at 18 months, demonstrating the biocompatibility of titanium alloy implants.

Postoperative swelling caused instability of the patella in full flexion, similar to a first-degree residual patellar luxation. This condition gradually resolved approximately one month after surgery, with resolution of the knee swelling and muscle alignment. In all cases, weight bearing was observed two days after surgery. Postoperative monitoring aims to guide the patient towards full functional recovery of the limb through a physiotherapy program for optimal results, as evidenced by the follow-up of treated patients. Physiotherapy and rehabilitation following orthopedic surgery have become common practice in veterinary orthopedics for full return to normal activity and prevention of secondary joint and muscle disease such as ankylosis or acquired muscle contractures [56,57,58]. A physiotherapy program is essential for complete limb recovery in dogs with grade 4 PL associated with severe quadriceps contracture. Postoperative PROM and stretching programs are particularly important when loss of motion is present or anticipated and should be implemented as soon as loss of joint motion is identified, as limb disuse and periarticular fibrosis tend to be self-sustaining [56].

Pelvic limb disuse following femoral ostectomy, usually caused by the combination of severe pain on extension and lack of it, is the most common clinical situation that warrants a stretching program. As extension increases, the residual pain felt at full extension is outside the PROM used in walking and trotting, allowing the patient to strengthen the limb through exercise [56,57]. Physiotherapy played an important role in resolving the contracture of the quadriceps mechanism, gradually reducing the dorsal kyphosis and inflammation and improving PROM of the stifle joint.

The result of freehand corrective osteotomies can be challenging because the position, orientation and extent of the osteotomy depends on the surgeon’s intraoperative judgement [23,59,60]. Intraoperative implementation of preoperative angular and torsional corrections is challenging and requires intraoperative measurements and imaging studies [27]. The use of the tibial plateau levelling osteotomy jig to provide temporary stability to the distal femoral osteotomy while maintaining limb alignment in the frontal and axial planes prior to internal plate fixation of the osteotomy was described by [61]. The tibial plateau levelling osteotomy jig provides only relative stability after osteotomy, so it is usually used in combination with bone reduction forceps or divergent Kirschner wires prior to bone-plate fixation. These factors increase the risk of surgeon-induced deformity and/or undercorrection or overcorrection of the femoral deformity. When using the Deformity Reduction Device (DRD), the bone plate is applied with the osteotomy stabilized by two pins proximal and two pins distal to the osteotomy site, with no need for an assistant or reduction forceps to maintain the reduction. The amount of angulation and torsional correction can be directly visualized on the graduated scales of the arch and hinge [15,16,61].

The integration of VSP and PSI into veterinary medicine opens up new frontiers in veterinary orthopedics, traumatology and neurosurgery. The use of these technologies for the design and manufacture of patient-specific orthopedic devices has proven to be extremely advantageous, as it can offer individualized solutions that can be adapted to the specific needs of the patient [23,24]. These tools can address a wide range of complex and difficult-to-solve orthopedic conditions, reducing intraoperative time, improving surgical precision and contributing to rapid functional recovery. The development of 3D printing and the use of advanced materials offer even broader perspectives to further explore and promote innovation in veterinary medicine to improve animal care and welfare.

This study has some limitations including the small number of cases. Future controlled prospective case studies would be beneficial to determine the potential superiority of this technique over traditional planning and surgical modalities in managing dogs with patellar laxation secondary to limb deformity. Finally, there are production costs associated with performing PSI, making the procedure more expensive for the owner and longer waiting times for surgery. Waiting times for PSIs such as patient-specific guides and plates depend on both the surgeon and the bioengineer, as well as the company and correspond to the design, production and shipping phases of the final product. Therefore, the relationship between the surgeon and the bioengineer and the turnaround time become very important when designing a patient-specific osteotomy guide to save time. Usually, it takes three to four weeks from the CT scan to the arrival of the guide and plate [62,63]. In our study, the expected waiting time for the arrival of the PSI and thus for the performance of corrective surgery, was three weeks from VSP. In the opinion of the authors, the increase in waiting time and cost of the procedure reported in this study was justified by the increase in surgical precision, the reduction in operating time and, consequently, the cost of anesthesia. In addition, the reduction in functional recovery time due to the use of PSI leads to an increase in animal welfare and owner satisfaction.

## 4. Conclusions

In our study, virtual surgical planning (VSP) combined with the use of patient-specific 3D-printed osteotomy guides and titanium plates led to optimal outcomes in both patients. Using these technologies, the distal femoral osteotomy (DFO) and subsequent plate fixation ensured accurate correction of femoral deformities. Patient-specific osteotomy guides reduced intraoperative time, eliminated the need for intraoperative diagnostics and ensured a more accurate osteotomy. Patient-specific titanium plates reduced surgical complications and allowed for shorter postoperative recovery times. Looking forward, it is important to highlight how collaboration between two different professionals, such as the bioengineer and the surgeon, can improve the accuracy of corrective osteotomies, reduce the complication rate and consequently improve the animal’s quality of life.

## Figures and Tables

**Table 1:** aLDFA(°): anatomical latero-distal femoral angle; Proc: procurvatum; FTA: femoral torsion angle; Pre Op: Preoperative; Virtual Op: virtual operative; Post Op: Postoperative; CWO: closed wedge osteotomy; TBR: trochlear block recession; LD: Lateral desmotomy; MER: medial imbrication of retinaculum.

Dog	Side	aLDFA(°)			Proc.			FTA			Imaging Modality	Concurrent Deformity	Other Procedures
		Pre Op	Virtual Op	Post Op	Pre Op	Virtual Op	Post Op	Pre Op	Virtual Op	Post Op			
1	R	83°	94°	98°	158°	170°	170°	25°	20°	20°	CT	Mild Metatarsal rotation	CWO; TBR; LD; MER.
1	L	82°	94°	98°	156°	170°	169°	29°	20°	20°	CT	Mild Metatarsal rotation	CWO; TBR; LD; MER.
2	R	82°	94°	95°	163°	170°	172°	29°	20°	20°	CT	Mild Metatarsal rotation	CWO; TBR; LD; MER.

**Table 2:** PROM: passive range of motion; TIE: circumference at the level of the greater trochanter; FWUP: Follow-up; m: months. 1: stifle extension angle; 2: stifle flexion angle.

		PROM					TIE Circumference				
Dog	Side	Pre Operative	FWUP 6 m	FWUP 18 m	FWUP 24 m	FWUP 36 m	Pre Operative	FWUP 6 m	FWUP 18 m	FWUP 24 m	FWUP 36 m
1	R	1 = 130 2 = 35°	1 = 140 2 = 90°	1 = 150 2 = 80°	1 = 138 2 = 80°	1 = 138 2 = 80°	28 cm	38 cm	42 cm	42 cm	42 cm
1	L	1 = 125 2 = 35°	1 = 140 2 = 80°	1 = 138 2 = 80°	1 = 138 2 = 80°	1 = 140 2 = 80°	28 cm	39 cm	32 cm	39 cm	40 cm
2	R	1 = 130 2 = 35°	1 = 155 2 = 70°	1 = 155 2 = 60°	1 = 155 2 = 60°	1 = 155 2 = 60°	30 cm	37 cm	42 cm	42 cm	42 cm

## Data Availability

The data presented in this study are available on justified request from the corresponding author.

## References

[1] Di Dona F., Della Valle G., Fatone G. (2018). Patellar luxation in dogs. Vet. Med..

[2] Di Dona F., Della Valle G., Balestriere C., Lamagna B., Meomartino L., Napoleone G., Lamagna F., Fatone G. (2016). Lateral patellar luxation in nine small breed dogs. Open Vet. J..

[3] Kalff S., Butterworth S.J., Miller A., Keeley B., Baines S., McKee W.M. (2014). Lateral patellar luxation in dogs: A retrospective study of 65 dogs. Vet. Comp. Orthop. Traumatol..

[4] Shaver S.L., Mayhew K.N., Sutton J.S., Mayhew P.D., Runge J.J., Brown D.C., Kass P.H. (2014). Complications after corrective surgery for lateral patellar luxation in dogs: 36 cases (2000–2011). J. Am. Vet. Med. Assoc..

[5] Gibbons S.E., Macias C., Tonzing M.A., Pinchbeck G.L., McKee W.M. (2006). Patellar luxation in 70 large breed dogs. J. Small Anim. Pract..

[6] Alam M.R., Lee J.I., Kang H.S., Kim I.S., Park S.Y., Lee K.C., Kim N.S. (2007). Frequency and distribution of patellar luxation in dogs. 134 cases (2000 to 2005). Vet. Comp. Orthop. Traumatol..

[7] LaFond E., Breur G.J., Austin C.C. (2002). Breed susceptibility for developmental orthopedic diseases in dogs. J. Am. Anim. Hosp. Assoc..

[8] L’Eplattenier H.F., Montavon P. (2002). Patellar luxation in dogs and cats: Management and prevention. Comp. Cont. Edu. Pract. Vet..

[9] Bound N., Zakai D., Butterworth S.J., Pead M. (2009). The prevalence of canine patellar luxation in three centres. Clinical features and radiographic evidence of limb deviation. Vet. Comp. Orthop. Traumatol..

[10] Harasen G. (2006). Patellar luxation: Pathogenesis and surgical correction. Can. Vet. J..

[11] DeTora M.D., Boudrieau R.J. (2016). Complex angular and torsional deformities (distal femoral malunions). Preoperative planning using stereolithography and surgical correction with locking plate fixation in four dogs. Vet. Comp. Orthop. Traumatol..

[12] Arthurs G.I., Langley-Hobbs S.J. (2006). Complications associated with corrective surgery for patellar luxation in 109 dogs. Vet. Surg..

[13] Roch S.P., Gemmill T.J. (2008). Treatment of medial patellar luxation by femoral closing wedge ostectomy using a distal femoral plate in four dogs. J. Small Anim. Pract..

[14] Bosio F., Bufalari A., Peirone B., Petazzoni M., Vezzoni A. (2017). Prevalence, treatment and outcome of patellar luxation in dogs in Italy. A retrospective multicentric study (2009–2014). Vet. Comp. Orthop. Traumatol..

[15] Brower B.E., Kowaleski M.P., Peruski A.M., Pozzi A., Dyce J., Johnson K.A., Boudrieau R.J. (2017). Distal femoral lateral closing wedge osteotomy as a component of comprehensive treatment of medial patellar luxation and distal femoral varus in dogs. Vet. Comp. Orthop. Traumatol..

[16] Panichi E., Cappellari F., Olimpo M., Piras L.A., Radasch R., Ferretti A., Peirone B. (2016). Distal femoral osteotomy using a novel deformity reduction device. Vet. Comp. Orthop. Traumatol..

[17] Swiderski J.K., Palmer R.H. (2007). Long-term outcome of distal femoral osteotomy for treatment of combined distal femoral varus and medial patellar luxation: 12 cases (1999–2004). J. Am. Vet. Med. Assoc..

[18] Gigi R., Gortzak Y., Barriga Moreno J., Golden E., Gabay R., Rumack N., Yaniv M., Dadia S., Segev E. (2022). 3D-printed Cutting Guides for Lower Limb Deformity Correction in the Young Population. J. Pediatr. Orthop..

[19] Oraa J., Beitia M., Fiz N., González S., Sánchez X., Delgado D., Sánchez M. (2021). Custom 3D-Printed Cutting Guides for Femoral Osteotomy in Rotational Malalignment Due to Diaphyseal Fractures: Surgical Technique and Case Series. J. Clin. Med..

[20] Fiz N., Delgado D., Sánchez X., Sánchez P., Bilbao A.M., Oraa J., Sánchez M. (2017). Application of 3D Technology and Printing for Femoral Derotation Osteotomy: Case and Technical Report. Ann. Transl. Med..

[21] Wong K.C. (2016). 3D-Printed Patient-Specific Applications in Orthopedics. Orthop. Res. Rev..

[22] Chai W., Xu M., Zhang G., Zhang L., Gou W., Ni M., Chen J. (2013). Computer-Aided Design and Custom-Made Guide in Corrective Osteotomy for Complex Femoral Deformity. J. Huazhong Univ. Sci. Technol. Med. Sci..

[23] Memarian P., Pishavar E., Zanotti F., Trentini M., Camponogara F., Soliani E., Gargiulo P., Isola M., Zavan B. (2022). Active Materials for 3D Printing in Small Animals: Current Modalities and Future Directions for Orthopedic Applications. Int. J. Mol. Sci..

[24] Mendaza-DeCal R., Peso-Fernandez S., Rodriguez-Quiros J. (2023). Orthotics and prosthetics by 3D-printing: Accelerating its fabrication flow. Res. Vet. Sci..

[25] De Armond C.C., Lewis D.D., Kim S.E., Biedrzycki A.H. (2022). Accuracy of virtual surgical planning and custom three-dimensionally printed osteotomy and reduction guides for acute uni- and biapical correction of antebrachial deformities in dogs. J. Am. Vet. Med. Assoc..

[26] Carvajal J.L., Kim S.E. (2023). Proximal femoral deformity correction and total hip arthroplasty in a dog using 3D printed custom guides. Vet. Surg..

[27] Carwardine D.R., Gosling M.J., Burton N.J., O’Malley F.L., Parsons K.J. (2021). Three-dimensional-printed patient-specific osteotomy guides, repositioning guides and titanium plates for acute correction of antebrachial limb deformities in dogs. Vet. Comp. Orthop. Traumatol..

[28] Hall E.L., Baines S., Bilmont A., Oxley B. (2019). Accuracy of patientspecific three-dimensional-printed osteotomy and reduction guides for distal femoral osteotomy in dogs with medial patella luxation. Vet. Surg..

[29] Oxley B. (2017). Bilateral shoulder arthrodesis in a Pekinese using three-dimensional printed patient-specific osteotomy and reduction guides. Vet. Comp. Orthop. Traumatol..

[30] Fracka A.B., Oxley B., Allen M.J. (2023). 3D-printed, patient-specific cutting guides improve femoral and tibial cut alignment in canine total knee replacement. Vet. Surg..

[31] Longo F., Penelas A., Gutbrod A., Pozzi A. (2019). Three-dimensional computer-assisted corrective osteotomy with a patient-specific surgical guide for an antebrachial limb deformity in two dogs. Schweiz. Arch. Tierheilkd..

[32] Longo F., Nicetto T., Knell S.C., Evans R.B., Isola M., Pozzi A. (2022). Three-dimensional volume rendering planning, surgical treatment, and clinical outcomes for femoral and tibial detorsional osteotomies in dogs. Vet. Surg..

[33] Shi J., Lv W., Wang Y., Ma B., Cui W., Liu Z., Han K. (2019). Three dimensional patient-specific printed cutting guides for closing-wedge distal femoral osteotomy. Int. Orthop..

[34] Worth A.J., Crosse K.R., Kersley A. (2019). Computer-Assisted Surgery Using 3D Printed Saw Guides for Acute Correction of Antebrachial Angular Limb Deformities in Dogs. Vet. Comp. Orthop. Traumatol..

[35] Popov V.V., Muller-Kamskii G., Katz-Demyanetz A., Kovalevsky A., Usov S., Trofimcow D., Dzhenzhera G., Koptyug A. (2019). Additive manufacturing to veterinary practice: Recovery of bony defects after the osteosarcoma resection in canines. Biomed. Eng. Lett..

[36] Easter T.G., Bilmont A., Pink J., Oxley B. (2020). Accuracy of threedimensional printed patient-specific drill guides for treatment of canine humeral intracondylar fissure. Vet. Surg..

[37] Lam G., Kim S.Y. (2018). Three-dimensional computer-assisted surgical planning and use of three-dimensional printing in the repair of a complex articular femoral fracture in a dog. Vet. Comp. Orthop. Traumatol..

[38] Oxley B. (2018). A 3-dimensional-printed patient-specific guide system for minimally invasive plate osteosynthesis of a comminuted mid-diaphyseal humeral fracture in a cat. Vet. Surg..

[39] Toni C., Oxley B., Behr S. (2020). Atlanto-axial ventral stabilisation using 3D-printed patient-specific drill guides for placement of bicortical screws in dogs. J. Small Anim. Pract..

[40] Kamishina H., Sugawara T., Nakata K., Nishida H., Yada N., Fujioka T., Nagata Y., Doi A., Konno N., Uchida F. (2019). Clinical application of 3D printing technology to the surgical treatment of atlantoaxial subluxation in small breed dogs. PLoS ONE.

[41] Toni C., Oxley B., Clarke S., Behr S. (2021). Accuracy of placement of pedicle screws in the lumbosacral region of dogs using 3Dprinted patient-specific drill guides. Vet. Comp. Orthop. Traumatol..

[42] Hamilton-Bennett S.E., Oxley B., Behr S. (2018). Accuracy of a patient-specific 3D printed drill guide for placement of cervical transpedicular screws. Vet. Surg..

[43] Dudley R.M., Kowaleski M.P., Drost W.T., Dyce J. (2006). Radiographic and computed tomographic determination of femoral varus and torsion in the dog. Vet. Radiol. Ultrasound.

[44] Paley D., Paley D., Herzenberg J.E. (2002). Principles of Deformity Correction.

[45] Kieves N.R., Bergh M.S., Zellner E., Wang C. (2016). Pilot study measuring the effects of bandaging and cold compression therapy following tibial plateau levelling osteotomy. J. Small Anim. Pract..

[46] Drygas K.A., Mcclure S.R., Goring R.L., Pozzi A., Robertson S.A., Wang C. (2011). Effect of cold compression therapy on postoperative pain, swelling, range of motion, and lameness after tibial plateau leveling osteotomy in dogs. J. Am. Vet. Med. Assoc..

[47] Szabo S.D., Levine D., Marcellin-Little D.J., Sidaway B.K., Hofmeister E., Urtuzuastegui E. (2020). Cryotherapy Improves Limb Use but Delays Normothermia Early After Stifle Joint Surgery in Dogs. Front. Vet. Sci..

[48] Fox D.B., Tomlinson J.L., Cook J.L., Breshears L.M. (2006). Principles of uniapical and biapical radial deformity correction using dome osteotomies and the center of rotation of angulation methodology in dogs. Vet. Surg..

[49] Knapp J.L., Tomlinson J.L., Fox D.B. (2016). Classification of angular limb deformities affecting the canine radius and ulna using the center of rotation of angulation method. Vet. Surg..

[50] Meola S.D., Wheeler J.L., Rist C.L. (2008). Validation of a technique to assess radial torsion in the presence of procurvatum and valgus deformity using computed tomography: A cadaveric study. Vet. Surg..

[51] Piras L.A., Peirone B., Fox D. (2012). Effects of antebrachial torsion on the measurement of angulation in the frontal plane: A cadaveric radiographic analysis. Vet. Comp. Orthop. Traumatol..

[52] Eby A., Bleedorn J. (2020). A computed tomographic graphical approach to guide correction of femoral torsion. Vet. Surg..

[53] Oxley B., Gemmill T.J., Pink J., Clarke S., Parry A., Baines S., Malcolm McKee W. (2013). Precision of a novel computed tomographic method for quantification of femoral varus in dogs and an assessment of the effect of femoral malpositioning. Vet. Surg..

[54] Barnes D.M., Anderson A.A., Frost C., Barnes J. (2015). Repeatability and reproducibility of measurements of femoral and tibial alignment using computed tomography multiplanar reconstructions. Vet. Surg..

[55] Tomlinson J., Fox D., Cook J.L., Keller G.G. (2007). Measurement of femoral angles in four dog breeds. Vet. Surg..

[56] Marcellin-Little D.J., Levine D. (2015). Principles and application of range of motion and stretching in companion animals. Vet. Clin. N. Am. Small Anim. Pract..

[57] Baltzer W.I. (2020). Rehabilitation of companion animals following orthopaedic surgery. N. Z. Vet. J..

[58] Drum M.G., Marcellin-Little D.J., Davis M.S. (2015). Principles and applications of therapeutic exercises for small animals. Vet. Clin. N. Am. Small Anim. Pract..

[59] Townsend A., Guevar J., Oxley B., Hetzel S., Bleedorn J. (2024). Comparison of three-dimensional printed patient-specific guides versus freehand approach for radial osteotomies in normal dogs: Ex vivo model. Vet. Surg..

[60] Lee H.R., Adam G.O., Yang D.K., Tungalag T., Lee S.J., Kim J.S., Kang H.S., Kim S.J., Kim N.S. (2020). An Easy and Economical Way to Produce a Three-Dimensional Bone Phantom in a Dog with Antebrachial Deformities. Animals.

[61] Olimpo M., Piras L.A., Peirone B., Fox D.B. (2017). Comparison of osteotomy technique and jig type in completion of distal femoral osteotomies for correction of medial patellar luxation: An in vitro study. Vet. Comp. Orthop. Traumatol..

[62] Hespel A.M., Wilhite R., Hudson J. (2014). Invited review—Applications for 3D printers in veterinary medicine. Vet. Radiol. Ultrasound.

[63] Mulford J.S., Babazadeh S., Mackay N. (2016). Three-dimensional printing in orthopaedic surgery: Review of current and future applications. ANZ J. Surg..

